# Evaluation of the IOTA ADNEX Model, Two‐Step Strategy and RMI in Routine Gynaecologic Care in Denmark and Implications for Implementation: A Prospective Multicenter Cohort Study

**DOI:** 10.1111/1471-0528.70253

**Published:** 2026-04-26

**Authors:** Nikoline Schou Karlsen, Eva Dreisler, Claus Kim Høgdall, Estrid Solyom Høgdall, Mona Aarenstrup Karlsen, Thomas Alexander Gerds, Guðbjörg Andrésdóttir, Lea Langhoff‐Thuesen, Malene Merete Forstholm, Abelone Elisabeth Sakse

**Affiliations:** ^1^ Department of Obstetrics and Gynecology Copenhagen University Hospital, Rigshospitalet Copenhagen Denmark; ^2^ Molecular Unit, Department of Pathology Herlev & Gentofte University Hospital Herlev Denmark; ^3^ Institute of Clinical Medicine University of Copenhagen Faculty of Health and Medical Sciences Copenhagen Denmark; ^4^ Department of Public Health, Section of Biostatistics University of Copenhagen Copenhagen Denmark; ^5^ Department of Obstetrics and Gynecology North Zealand University Hospital Hillerød Denmark; ^6^ Department of Obstetrics and Gynecology Amager & Hvidovre University Hospital Hvidovre Denmark; ^7^ Department of Obstetrics and Gynecology Herlev & Gentofte University Hospital Herlev Denmark

**Keywords:** adnexal masses, diagnostic models, IOTA ADNEX model, ovarian cancer, referral thresholds, risk of malignancy index

## Abstract

**Objective:**

To evaluate whether the IOTA ADNEX model and the Two‐Step Strategy improve triage and referral of adnexal masses in routine gynaecologic care compared with the RMI and to identify an appropriate malignancy‐risk threshold.

**Design:**

Prospective multicenter cohort study.

**Setting:**

Thirteen non‐tertiary hospitals and clinics and one tertiary referral hospital in Denmark.

**Sample:**

A complete‐case cohort of 966 patients with adnexal masses.

**Methods:**

Malignancy risk was estimated using prospectively collected clinical data, ultrasound findings, and CA125 levels. Reference standard was histopathology or ≥ 12 months of clinical follow‐up. Performance was evaluated across predefined thresholds (1%–30% for ADNEX/Two‐Step Strategy (modified benign descriptors + ADNEX); ≥ 200 for RMI), stratified by centre type.

**Outcome Measures:**

Negative and positive predictive values (NPV, PPV), sensitivity, referral rates to assess correct and incorrect referrals.

**Results:**

In non‐tertiary centres, NPVs were ≥ 96% for IOTA models versus 95% for RMI; corresponding values in the tertiary centre were 82%–100% versus 78%. PPVs increased with higher thresholds and approached RMI at 20% threshold. In non‐tertiary centres, where referral decisions are made, a 15% threshold provided the most favourable balance between sensitivity (~63%) and referral rate (~14%). At thresholds ≥ 25%, referral rates were similar to RMI (~8%), with only marginal gains in sensitivity (~50% vs. 39%). Most additionally detected tumours were stage I ovarian cancers or borderline tumours. For masses classified as benign by modified benign descriptors, ADNEX showed high NPVs but low PPVs and negligible net benefit, providing limited additional diagnostic value over the Two‐Step Strategy.

**Conclusions:**

IOTA‐based models improve early detection but increase referral rates. A 15% risk threshold appears to offer a clinically reasonable balance between early detection of malignancy and referral burden, based on observed trade‐offs between detection and referral rates.

**Trial Registration:**

ClinicalTrials.gov identifier: NCT04188652

## 1 Introduction

Timely and accurate triage of adnexal masses is crucial. Patients with suspected ovarian cancer (OC) should be referred to specialized gynaecologic oncology centres, where management is associated with improved overall survival, particularly in advanced‐stage disease [1, 2, 3]. Those with likely benign disease can be safely managed in general gynaecology units [1, 2, 3, 4]. Inaccurate triage may delay referral in patients with malignancy or result in unnecessary investigations, extensive surgery, patient anxiety and increased healthcare utilization [5, 6].

Denmark has one of the highest incidence rates among the Nordic countries, with approximately 550 patients diagnosed annually (age‐standardized incidence rate of 15 per 100 000) [7, 8]. Around 70% present with advanced‐stage disease [7, 8, 9]. Prognosis is strongly stage‐dependent, with 5‐year survival exceeding 90% in stage I but falling below 20% in stage IV [7, 8, 9].

In Denmark, OC management is centralized to three highly specialized gynaecologic oncology centres. The diagnostic work‐up of suspected OC follows a structured national fast‐track cancer pathway with defined target time intervals from referral to diagnosis and treatment initiation. Patients with suspected adnexal pathology are referred by their general practitioner for gynaecologic evaluation in hospital‐based departments or outpatient clinics. Transvaginal ultrasound is performed at the point of care by the gynaecologist, and the Risk of Malignancy Index (RMI) is calculated [10, 11, 12, 13]. An RMI ≥ 200 primarily determines referral to specialized gynaecologic oncology centres for further management [10, 11, 12, 13].

The International Ovarian Tumour Analysis (IOTA) Assessment of Different NEoplasias in the adneXa (ADNEX) model [14] is recommended in international consensus statements [15, 16]. It has demonstrated higher discriminative ability (area under the receiver operating characteristic curve (AUC) and sensitivity at clinically relevant thresholds) than the RMI in several validation studies and meta‐analyses [17, 18, 19, 20]. However, performance varies across study populations and clinical settings. To facilitate clinical implementation, the Two‐Step Strategy was proposed [21]. This approach identifies clearly benign tumours using modified Benign Descriptors (BD), thereby reducing the need for ADNEX model calculations and streamlining clinical workflow while maintaining diagnostic performance [21, 22, 23, 24].

Despite these findings, evidence from prospective, multicenter studies conducted in routine clinical practice, where ultrasound is performed by the examining clinician, remains limited [17, 18, 19, 20, 24, 25, 26]. In Denmark, IOTA certification is not mandatory, and the use of standardized IOTA terminology is not consistent in routine clinical practice. In addition, no consensus exists regarding the most appropriate threshold for referral to tertiary gynaecological oncology centres [17, 18, 19, 20]. Implementation within the Danish healthcare system requires evidence on how model performance varies across clinically relevant malignancy risk thresholds to support triage decisions that balance missed malignancies against unnecessary referrals within available resources.

This study aimed to evaluate the diagnostic performance of the IOTA ADNEX model and the Two‐Step Strategy with modified BD compared with RMI currently used in Denmark. Secondary objectives were to evaluate clinically relevant malignancy‐risk thresholds for referral to gynaecological oncology centres and to assess the potential impact of implementing these models in routine clinical care.

## 2 Methods

### 2.1 Study Design and Participants

This was a prospective, multicenter, observational study conducted at nine general gynaecology practices and five gynaecological departments located at four general hospitals and one tertiary referral hospital (Rigshospitalet, North Zealand Hospital, Herlev Hospital, Hvidovre Hospital and Holbæk Hospital). The tertiary referral hospital included units offering highly specialized gynaecologic services, including a gynaecologic oncology unit, alongside a general gynaecology unit serving the local population. General gynaecology practices in Denmark operate within the publicly funded healthcare system and are integrated into the national referral pathway. In this setting, practicing gynaecologists in specialist clinics and general gynaecologists in hospital‐based departments have comparable roles in the diagnostic evaluation of adnexal masses.

Patients aged ≥ 18 years with at least one adnexal mass on ultrasound were eligible. Adnexal masses were defined according to IOTA criteria as structures inconsistent with normal ovarian physiological function [27]. Pregnant participants were not excluded a priori. Exclusion criteria were age < 18 years, ovarian structures consistent with normal ovarian physiological function, or withdrawal of informed consent.

The examining clinicians, either a gynaecologist or a supervised specialty trainee, assessed eligibility, made all clinical management decisions in accordance with Danish national guidelines [11] and provided the oral and written study information after the routine consultation. All participants subsequently gave oral and written informed consent. Recruitment was prospective but not strictly consecutive, as inclusion depended on clinical staff during routine care. Ethical approval did not permit screening of outpatient schedules or medical records prior to consent, and we therefore only had information on patients who agreed to participate. Recruitment began in January 2020 at the tertiary referral hospital. Non‐tertiary sites joined stepwise during 2022, and by 2023 recruitment was active at all participating centres. Recruitment at the non‐tertiary sites was expanded due to COVID‐19‐related and logistical delays. The study was approved by the Danish Data Protection Agency (P‐2019‐340) and the Ethics Committee (H‐19021342) and was registered at ClinicalTrials.gov (entry date: 11‐01‐2019).

### 2.2 Data Collection and Reference Standard

All study data were prospectively collected, and included clinical information, ultrasound descriptions and biobank blood samples. At inclusion, examining clinicians prospectively recorded clinical information, diagnostic findings and management in the electronic patient record as part of routine care. Postmenopausal status was defined as ≥ 12 months of spontaneous amenorrhea in women aged ≥ 45 years, or age ≥ 50 years in women with a prior hysterectomy. All electronic medical records were reviewed by December 2024, ensuring at least 12 months of follow‐up for all participants. For participants with a histopathological diagnosis, the reference standard was based on tissue or cytology results, including specimens obtained by surgery, biopsy or cytology, with pathologists blinded to clinical data and ultrasound findings. Linkage to the Danish National Pathology Registry ensured capture of all histopathological or cytologically verified diagnoses during follow‐up. For participants without a histopathological diagnosis, who were managed conservatively, benign classification was based on clinical follow‐up documented in the medical records, including resolution, stability or regression of the mass on imaging and absence of clinical suspicion of malignancy.

### 2.3 Ultrasound Assessment

Ultrasound examinations were performed transvaginally, or transabdominally when indicated, by a gynaecologist or a supervised specialty trainee as part of routine clinical practice. Both groups comprised IOTA‐certified and non‐IOTA‐certified clinicians. IOTA certification status was verified through the official IOTA website [28]. Clinicians were instructed to describe the morphological features and measurements in accordance with the IOTA definitions and terminology [27]. Ultrasound findings were documented in the using a study template integrated into local electronic patient record systems.

Plenary sessions and one‐to‐one guidance were conducted at each site before and during recruitment to review the study protocol and IOTA terminology. Educational IOTA materials were available in all ultrasound rooms [29]. No quality assessment of ultrasound images or descriptions was performed during data collection, and no structured image review sessions were held, in accordance with the routine clinical practice.

### 2.4 CA125 Collection

CA125 levels were measured from biobank samples collected upon inclusion and processed per Danish Cancer Biobank guidelines [30]. CA125 was analysed using the Elecsys CA125 II assay on a cobas e 801 analyser (Roche Diagnostics) at Rigshospitalet [31]. If no biobank sample was available, the most recent routine CA125 result within 3 months prior to inclusion was used.

### 2.5 Risk Prediction Models

As RMI ≥ 200 was the nationally recommended triage tool during the study recruitment period, clinicians were not instructed to apply IOTA risk models to avoid clinical decisions being influenced by research‐related risk estimates. Thus, ADNEX and Two‐Step Strategy risks were calculated retrospectively after data collection was complete, using prospectively collected clinical data and clinician‐reported ultrasound descriptions. When ultrasound variables were not explicitly documented, features such as solid components, papillary projections, acoustic shadows or ascites were interpreted as absent. Thus, no imputation of missing ultrasound variables was performed. If multiple masses were present, the largest or most complex lesion was selected. For analyses, tumours were grouped into the five ADNEX categories and dichotomized as benign or malignant (borderline, stage I–IV OC, or metastatic non‐ovarian malignancy), according to the model's definition of overall malignancy risk. Published formulas were used to compute ADNEX risk estimates with CA125 [14], and analyses were restricted to the version including CA125, as comparing models with and without CA125 was beyond the scope of this study. Modified BDs were applied in the Two‐Step Strategy, following the approach described by Landolfo et al. [21]. Adnexal masses to which a modified BD was applicable were assigned a malignancy risk of 1% [21]. Otherwise, the ADNEX model was used for malignancy risk estimation. The Danish adaptation of RMI III was used [10, 32]. The RMI is calculated as the product of an ultrasound score, menopausal status and serum CA125 level (IU/mL). The ultrasound score is 1 (0–1 features) or 3 (≥ 2 features), based on multilocularity or bilocularity, solid components, bilaterality, papillary projections, ascites, or metastases. Menopausal status is scored as 1 for premenopausal and 3 for postmenopausal women.

### 2.6 Definition of ‘Oncology Center’ in ADNEX Risk Calculation

Participants examined at specialized tertiary care units within the tertiary referral hospital (Rigshospitalet) were classified as *oncology center = yes*. Participants examined at the general gynaecology unit (Rigshospitalet) or at the other non‐tertiary inclusion sites were classified as *oncology center = no*.

### 2.7 Statistical Analysis

A complete‐case analysis was performed including participants with available CA125 measurements and stored ultrasound images. CA125 measurements were obtained from either biobank samples collected at inclusion (77.1%) or from routine clinical measurements performed within 3 months prior to inclusion (22.9%). We compared the RMI (threshold ≥ 200) with the ADNEX model and the Two‐Step Strategy across prespecified thresholds (1%–30%). Thresholds were prespecified to reflect clinically relevant decision thresholds reported in previous studies and considered plausible for guiding referral in routine clinical practice. The aim was to identify clinically appropriate thresholds based on the trade‐off between detection of malignancy and avoidance of unnecessary referrals, relative to current clinical practice using the RMI (threshold ≥ 200). In this context, thresholds were evaluated by comparing sensitivity, predictive values and referral rates, reflecting the clinical consequences of different referral strategies. Diagnostic performance was assessed using negative and positive predictive values (NPV and PPV), and area under the receiver operating characteristic curve (AUC) . Confidence intervals (95%) were calculated for all performance metrics. Differences in AUC between models were compared using DeLong's test for correlated ROC curves. Prediction error (Brier scores) was calculated, and calibration plots are illustrated. Performance was stratified by centre type (non‐tertiary vs. tertiary) to account for differences in disease prevalence and case mix.

Threshold‐related analyses were conducted in participants from non‐tertiary centres, as they represent a routine‐care, pre‐triage population in which referral decisions are made. In contrast, most participants examined at the tertiary centre had already been referred based on RMI (post‐triage group). Predictive values, referral rates and malignancy detection were used to inform threshold recommendations. We also performed a sensitivity analysis using an expanded pre‐triage population, including both non‐tertiary centres and the specialized endometriosis unit within the tertiary hospital, as this unit represents a tertiary function without a primary oncologic referral role. Results are presented in the Supporting Information.

A pre‐specified subgroup analysis was conducted to compare ADNEX with the Two‐Step Strategy in adnexal masses where a modified BD was applicable, in order to assess whether ADNEX provided additional diagnostic value. In this subgroup, the Two‐Step Strategy classifies all such tumours as benign with a fixed 1% risk. At clinically relevant thresholds above 1%, this corresponds to no patients being referred, functionally equivalent to a ‘refer none’ strategy. NB was therefore used to evaluate the clinical utility of applying ADNEX compared with not referring patients in this subgroup. A positive NB indicates that use of ADNEX provides a net clinical benefit compared with a refer‐none strategy, taking into account the trade‐off between true positives and false positives. In addition, NPV and PPV were calculated to assess the diagnostic performance of ADNEX within this subgroup.

Pregnant participants were included in the primary analyses to represent routine clinical practice, as adnexal masses are also evaluated in this population. However, as the ADNEX model has not been specifically validated in pregnancy, a post hoc sensitivity analysis excluding pregnant participants (*n* = 18) was conducted to assess the robustness of the results. All analyses were conducted in R version 4.4.1 [33].

## 3 Results

**FIGURE 1:**
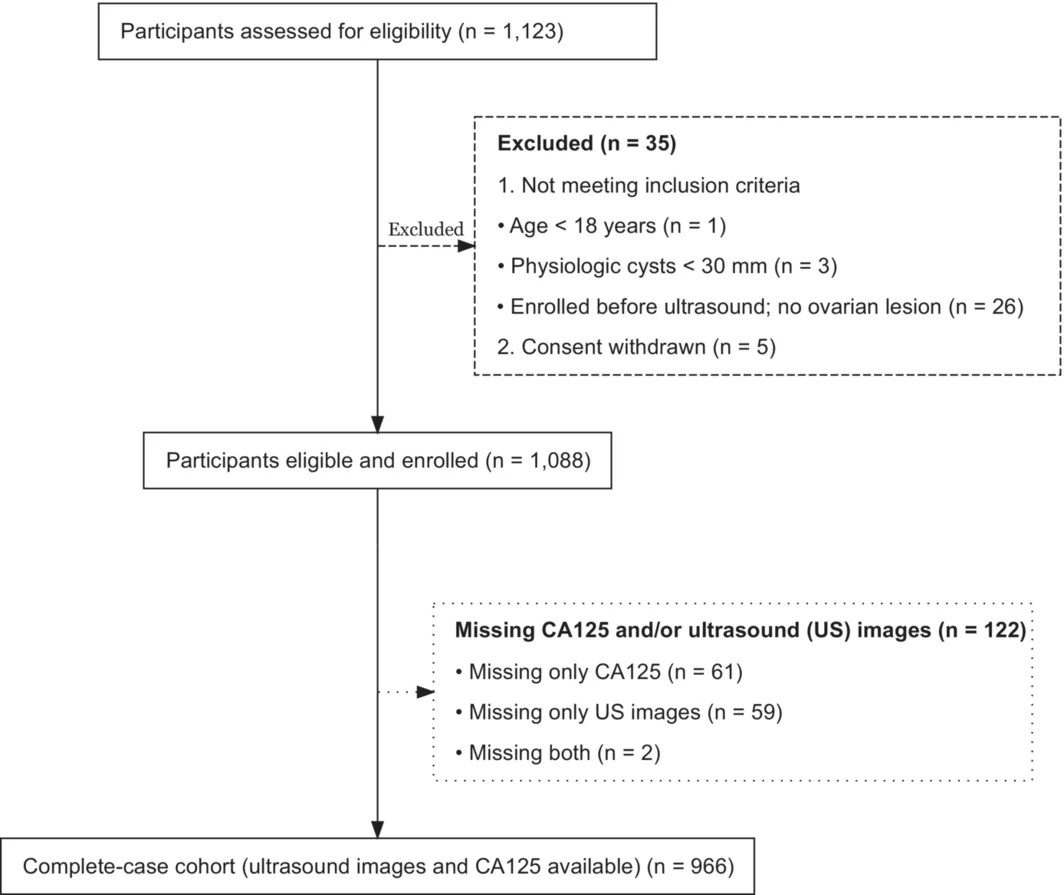
Flowchart of participant recruitment, inclusion and exclusion. A total of 1088 patients were eligible between 2020 and 2023. The complete‐case cohort (*n* = 966) included participants with available CA125 results and ultrasound images. All missing CA125 values were from benign cases, except one ovarian metastasis from disseminated gastric carcinoma.

From 2020 to 2023, 1088 patients were eligible for inclusion, of whom 966 (88.8%) had complete data and comprised the analytical cohort (Figure 1). Baseline characteristics and tumour outcomes by centre are shown in Table 1. Participants from the tertiary centre were older (median 54 vs. 51 years; *p* = 0.03) and had higher CA125 levels (median 54.7 vs. 14.4 U/mL; *p* < 0.001). Malignant or borderline tumours were more frequent in participants assessed at the tertiary centre (44% vs. 8%), consistent with differences in case mix between settings. A total of 122 participants (11.2%) were excluded from the complete‐case analysis due to missing CA125 measurements and/or unavailable ultrasound images. In this group, 82.0% had benign or likely benign tumours, 5.7% borderline tumours, 10.7% primary OC and 1.6% metastatic tumours.

**TABLE 1:** Baseline characteristics of the complete‐case population (*n* = 966), stratified by centre type.

Variable Presented as counts (n) and percentages (%), unless otherwise stated	Non‐tertiary centres^a^ (*n* = 587)	Tertiary centre^b^ (*n* = 379)	Total (*n* = 966)	*p*
Age (median [IQR])	51 [35, 64]	54 [38, 67]	52 [35, 65]	0.0317
CA125 (median [IQR])	14.4 [9.8, 22.4]	54.7 [27.8, 177.1]	20.6 [11.9, 53.3]	< 0.0001
Postmenopausal	288 (49.1)	208 (54.9)	496 (51.3)	0.0890
Pregnant participants	12 (2.0)	6 (1.6)	18 (1.9)	0.7842
Site of study enrollment				
Copenhagen university hospital	117 (19.9)	379 (100.0)	496 (51.4)	< 0.0001
North Zealand university hospital	148 (25.2)	—	148 (15.3)	
Hvidovre university hospital	118 (20.1)	—	118 (12.2)	
Herlev university hospital	65 (11.1)	—	65 (6.7)	
Holbæk university hospital	64 (10.9)	—	64 (6.6)	
General Gynaecology practices	75 (12.8)	—	75 (7.8)	
Examined by IOTA certified clinician	338 (57.6)	277 (73.1)	615 (63.7)	< 0.0001
Examined by a specialist in gynaecology (vs. trainees)	402 (68.5)	355 (93.1)	755 (78.2)	< 0.0001
Management strategy				
Surgery/further management^c^ < 120 days after inclusion	311 (53.0)	324 (85.5)	635 (65.7)	< 0.0001
Surgery after watchful waiting (> 120 days after inclusion)^d^	76 (13.0)	18 (4.8)	94 (9.8)	
End of care or discharge after watchful waiting (resolution/stable)	168 (28.7)	28 (7.4)	196 (20.3)	
Ongoing or referred watchful waiting^e^	32 (5.5)	9 (2.4)	41 (4.2)	
Maximal diameter of the lesion, mm	59 [44.1, 80.0]	81.3 [53, 118]	64 [47, 94]	< 0.0001
Maximal diameter of the largest solid part, mm	0 [0, 10]	15 [0, 46]	0 [0.0, 21.8]	< 0.0001
Number of papillary projections				
None	460 (78.4)	251 (66.2)	711 (73.6)	< 0.0001
One	87 (14.8)	51 (13.5)	138 (14.3)	
Two	22 (3.7)	25 (6.6)	47 (4.9)	
Three	7 (1.2)	20 (5.3)	27 (2.8)	
More than three	11 (1.9)	32 (8.4)	43 (4.5)	
More than 10 locules	26 (4.4)	55 (14.5)	81 (8.4)	< 0.0001
Acoustic shadows present	85 (14.5)	29 (7.7)	114 (11.8)	0.0019
Ascites present	10 (1.7)	53 (14.0)	63 (6.5)	< 0.0001
Benign	543 (92.5)	213 (6.2)	756 (78.3)	< 0.0001
Surgery	343 (58.4)	176 (46.4)	519 (53.7)	
Conservative	200 (34.1)	37 (9.8)	237 (24.5)	
Borderline	23 (3.9)	48 (12.7)	71 (7.3)	
Stage I ovarian cancer	9 (1.5)	27 (7.1)	36 (3.7)	
Stage II–IV ovarian cancer	7 (1.2)	66 (17.4)	73 (7.6)	
Metastatic cancer to the ovary	5 (0.9)	25 (6.6)	30 (3.1)	

*Note:* Data are presented as counts (%) for categorical variables and medians [IQR] for continuous variables.

^a^ Participants enrolled at general gynaecology units serving their local population across all inclusion sites.

^b^ Participants enrolled at Copenhagen university hospital, Rigshospitalet's specialized tertiary care units, comprising gynaecologic oncology and endometriosis services.

^c^ Further management also includes biopsy performed before neo‐ or adjuvant chemotherapy in cases of advanced malignancy or metastatic disease to the ovary.

^d^ Surgery performed after initial conservative management or watchful waiting.

^e^ Also includes participants discharged from hospital to a general gynaecology practice for continued watchful waiting outside the study setting; no subsequent follow‐up data were available within this cohort.

Predictive values were evaluated separately within non‐tertiary and tertiary settings, reflecting their different malignancy prevalences (Table 2). As expected, PPVs were higher and NPVs lower in the tertiary centre, with the opposite pattern observed in non‐tertiary centres. Across both settings, the ADNEX model and the Two‐Step Strategy achieved higher NPVs than RMI ≥ 200 at all thresholds (1%–30%) and lower PPVs up to approximately 25%. In the tertiary centre (malignancy prevalence 44%), NPVs for the ADNEX model and the Two‐Step Strategy ranged from 82% to 100%, compared with 78% for RMI ≥ 200. PPVs ranged from 45% to 61% at thresholds of 1%–10%, from 61% to 64% at 15%–20% and from 66% to 67% at thresholds ≥ 25%, compared with 65% for RMI ≥ 200. In non‐tertiary centres (malignancy prevalence 8%), NPVs for the ADNEX model and the Two‐Step Strategy were consistently high (≥ 96%), compared with 95% for RMI ≥ 200. PPVs ranged from 8% to 33% at thresholds of 1%–15%, increased to 37%–38% at 20%, and 46%–50% at thresholds ≥ 25%, compared with 39% for RMI ≥ 200.

**TABLE 2:** Diagnostic performance of the ADNEX model and Two‐Step Strategy across selected thresholds, stratified by centre type.

Threshold ≥	Model	PPV (95% CI)	NPV (95% CI)	Above threshold, *n* (%)	Malignant ≥ threshold, *n*/*N* (% of all malignant, 95% CI)
Non‐tertiary centres: *N* = 587 (60.8%). Malignancies *n* = 44 (7.5%)					
1%	ADNEX	8.9 (6.4–11.4)	100.0 (100.0–100.0)	494 (84.2%)	44/44 (100.0%; 92.0–100.0)
	Two‐step strategy	7.9 (5.6–10.1)	100.0 (100.0–100.0)	558 (95.1%)	44/44 (100.0%; 92.0–100.0)
5%	ADNEX	21.0 (15.1–26.8)	98.8 (97.7–99.8)	186 (31.7%)	39/44 (88.6%; 75.4–96.2)
	Two‐step strategy	22.1 (15.9–28.3)	98.6 (97.4–99.7)	172 (29.3%)	38/44 (86.4%; 72.6–94.8)
10%	ADNEX	26.4 (18.6–34.3)	97.4 (96.0–98.9)	121 (20.6%)	32/44 (72.7%; 57.2–85.0)
	Two‐step strategy	27.4 (19.2–35.7)	97.3 (95.8–98.7)	113 (19.3%)	31/44 (70.5%; 54.8–83.2)
15%	ADNEX	33.3 (23.3–43.4)	96.8 (95.3–98.4)	84 (14.3%)	28/44 (63.6%; 47.8–77.6)
	Two‐step strategy	33.3 (23.1–43.6)	96.6 (95.1–98.2)	81 (13.8%)	27/44 (61.4%; 45.5–75.6)
20%	ADNEX	37.7 (26.2–49.1)	96.5 (94.9–98.1)	69 (11.8%)	26/44 (59.1%; 43.2–73.7)
	Two‐step strategy	37.3 (25.7–48.9)	96.3 (94.7–98.0)	67 (11.4%)	25/44 (56.8%; 41.0–71.7)
25%	ADNEX	46.0 (32.2–59.8)	96.1 (94.4–97.7)	50 (8.5%)	23/44 (52.3%; 36.7–67.5)
	Two‐step strategy	45.8 (31.7–59.9)	95.9 (94.2–97.6)	48 (8.2%)	22/44 (50.0%; 34.6–65.4)
30%	ADNEX	50.0 (35.2–64.8)	95.9 (94.3–97.6)	44 (7.5%)	22/44 (50.0%; 34.6–65.4)
	Two‐step strategy	48.8 (33.9–63.8)	95.8 (94.1–97.5)	43 (7.3%)	21/44 (47.7%; 32.5–63.3)
200	RMI	38.6 (24.2–53.0)	95.0 (93.2–96.9)	44 (7.5%)	17/44 (38.6%; 24.4–54.5)
Tertiary centre: *N* = 379 (39.2%). Malignancies *n* = 166 (43.8%)					
1%	ADNEX	45.2 (40.1–50.3)	100.0 (100.0–100.0)	367 (96.8%)	166/166 (100%; 97.8–100.0)
	Two‐step strategy	44.7 (39.7–49.8)	100.0 (100.0–100.0)	371 (97.9%)	166/166 (100%; 97.8–100.0)
5%	ADNEX	52.8 (47.2–58.4)	94.4 (89.2–99.7)	307 (81.0%)	162/166 (97.6%; 93.9–99.3)
	Two‐step strategy	56.2 (50.4–62.0)	92.7 (87.5–97.9)	283 (74.7%)	159/166 (95.8%; 91.5–98.3)
10%	ADNEX	58.6 (52.6–64.6)	89.0 (83.3–94.6)	261 (68.9%)	153/166 (92.2%; 87.0–95.8)
	Two‐step strategy	60.6 (54.6–66.7)	88.5 (83.0–94.0)	249 (65.7%)	151/166 (91.0%; 85.5–94.9)
15%	ADNEX	60.6 (54.4–66.8)	85.5 (79.6–91.4)	241 (63.6%)	146/166 (88.0%; 82.0–92.5)
	Two‐step strategy	61.8 (55.6–68.0)	84.9 (79.1–90.7)	233 (61.5%)	144/166 (86.7%; 80.6–91.5)
20%	ADNEX	62.9 (56.6–69.3)	83.9 (78.1–89.7)	224 (59.1%)	141/166 (84.9%; 78.6–90.0)
	Two‐step strategy	64.1 (57.7–70.4)	83.3 (77.6–89.1)	217 (57.3%)	139/166 (83.7%; 77.2–89.0)
25%	ADNEX	63.6 (57.2–70.0)	82.7 (76.9–88.5)	217 (57.3%)	138/166 (83.1%; 76.6–88.5)
	Two‐step strategy	64.8 (58.3–71.2)	82.2 (76.5–88.0)	210 (55.4%)	136/166 (81.9%; 75.2–87.5)
30%	ADNEX	66.7 (60.2–73.1)	82.9 (77.3–88.4)	204 (53.8%)	136/166 (81.9%; 75.2–87.5)
	Two‐step strategy	67.0 (60.5–73.5)	82.1 (76.5–87.7)	200 (52.8%)	134/166 (80.7%; 73.9–86.4)
200	RMI	65.3 (58.5–72.0)	77.8 (71.9–83.7)	190 (50.1%)	124/166 (74.7%; 67.4–81.1)

*Note:* PPV and NPV refer to the proportions of correctly classified positive and negative cases. *Malignant*≥*threshold* shows the proportion of all malignant tumours detected at each threshold. Percentages in ‘≥ threshold’ columns are relative to the total study population. Percentages in ‘Malignant ≥ threshold’ columns are relative to all malignant tumours within each stratum (sensitivity).

In non‐tertiary centres, RMI ≥ 200 identified 39% (17 of 44) of malignancies with a referral rate of approximately 8% (44 of 587) (Table 2). At a 5% risk threshold, the IOTA models detected approximately 88% of malignancies, with referral rates around 30%, corresponding to roughly six additional referrals per additional malignancy detected compared with RMI. At a 10% threshold, sensitivity declined to approximately 72%, with referrals reduced to around 20% (approximately five additional referrals per malignancy). At a 15% threshold, sensitivity was approximately 63%, with a referral rate of around 14% (approximately three to four additional referrals per malignancy). At a 20% threshold, sensitivity dropped to approximately 59%, with referral rates of around 12% (approximately two to three additional referrals per malignancy). At thresholds ≥ 25%, referral rates (approximately 8%) were comparable to RMI ≥ 200, while sensitivity remained modestly higher at approximately 51% (22–23 of 44). Absolute numbers and confidence intervals for all thresholds are provided in Table 2. Confidence intervals for the sensitivity estimates (Table 2) widen at higher thresholds, indicating increased uncertainty due to the limited number of malignancies in non‐tertiary centres. Across thresholds, the additional malignancies detected by the ADNEX model and the Two‐Step Strategy compared with RMI were predominantly borderline ovarian tumours, with a smaller increase in stage I OC (e.g., at a 15% threshold for ADNEX: 65.2% (15 of 23) vs. 34.8% (8 of 23) borderline tumours and 55.6% (5 of 9) vs. 33.3% (3 of 9) stage I OC; Tables S1 and S2). Findings from our sensitivity analysis of an expanded pre‐triage population (*n* = 684), including non‐tertiary centres and a specialized tertiary endometriosis unit, were consistent with the main analysis (Table S3).

**FIGURE 2:**
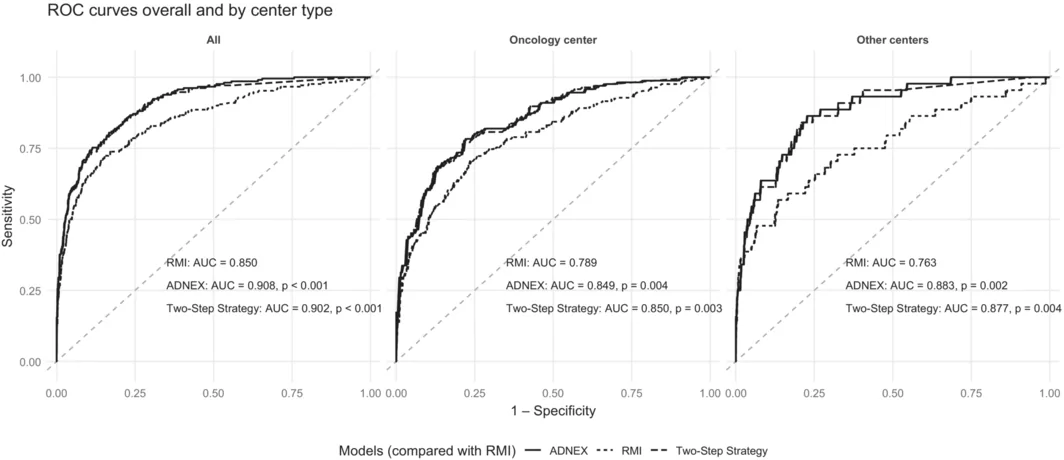
ROC curves for RMI, ADNEX model and Two‐Step Strategy, shown overall and stratified by centre type. AUC values and *p*‐values (vs. RMI) are presented within each panel.

Both the ADNEX model and the Two‐Step Strategy showed significantly higher discriminative ability than RMI across all settings (all *p* < 0.01) (Figure 2). In non‐tertiary centres, AUCs were 0.883 (95% CI 0.833–0.932) and 0.877 (95% CI 0.824–0.930) for ADNEX and the Two‐Step Strategy, respectively, compared with 0.763 (95% CI 0.676–0.849) for RMI. In the tertiary centre, corresponding AUCs were 0.849 (95% CI 0.810–0.887) and 0.850 (95% CI 0.812–0.888), compared with 0.789 (95% CI 0.742–0.835) for RMI. Prediction error was comparable for ADNEX and the Two‐Step Strategy within each stratum; higher in the tertiary centre (16.4 vs. 16.6) and lower in non‐tertiary centres (5.1 for both), which is likely attributable to the higher prevalence of malignancy and greater case complexity in tertiary settings rather than poorer intrinsic model performance. Calibration curves for both models showed a tendency toward slight underestimation in the risk range of approximately 5%–20% (Figure S1).

**FIGURE 3:**
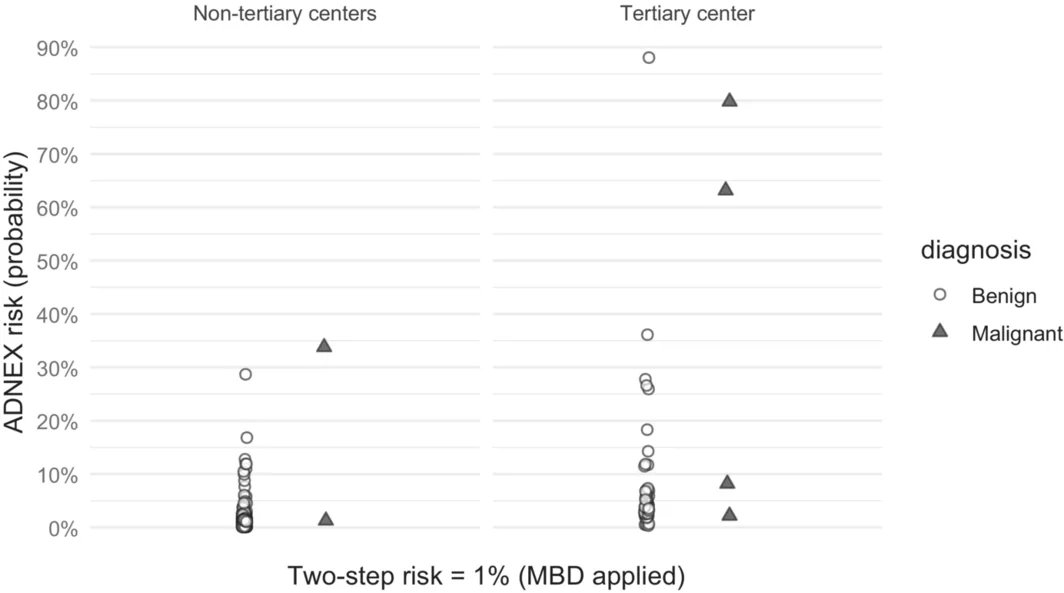
ADNEX‐predicted malignancy risks for adnexal masses (*n* = 300) classified as benign by the Two‐Step Strategy (modified BD applied; Two‐Step risk fixed at 1%), stratified by centre type. *Y*‐axis indicates ADNEX‐predicted risk (%). ○, Benign; ▲, malignant.

To assess the diagnostic value of the ADNEX model in masses classified as benign by the first step of the Two‐Step Strategy, we analysed the subgroup with an applicable modified benign descriptor (294 benign, five borderline, and one stage III low‐grade serous carcinoma) (Table 3). Given the small number of malignant and borderline tumours (*n* = 6), estimates should be interpreted with caution. In this subgroup, the ADNEX model showed high NPVs (≥ 96%–100%) across both non‐tertiary and tertiary centres, but low PPVs, ranging from 1%–33% in non‐tertiary and 7%–50% in tertiary centres, with wide confidence intervals. NB values were all positive and ≤ 0.004 in non‐tertiary centres and ≤ 0.055 in tertiary centres. These small NB values indicate only a minimal clinical advantage over a refer‐none strategy, as the Two‐Step Strategy effectively results in no referrals in this subgroup. This corresponds to at most a very small increase in correctly identified malignancies without substantially increasing unnecessary referrals. NB represents the balance between correctly identifying malignancies and avoiding unnecessary interventions. Figure 3 shows risk estimates for the subgroup: three borderline tumours had risks > 30%, two had risks of 8.2% and 1.8%, and the stage III low‐grade serous carcinoma had a risk of 2.2%. Raising the decision threshold from 10% to 30% reduced false positives without improving malignant detection.

**TABLE 3:** Diagnostic performance and clinical utility of the ADNEX model in masses with applicable Modified Benign Descriptors, stratified by centre type.

Threshold ≥	Non‐tertiary centres			Tertiary centre		
	PPV (95% CI)	NPV (95% CI)^a^	NB^b^	PPV (95% CI)	NPV (95% CI)^a^	NB^b^
1%	1.2 (0.0–2.7)	100.0 (100.0–100.0)	0.001	6.8 (0.4–13.2)	100.0 (100.0–100.0)	0.055
5%	7.1 (0.0–20.6)	99.6 (98.7–100.0)	0.001	12.5 (0.0–25.7)	97.4 (92.5–100.0)	0.030
10%	12.5 (0.0–35.4)	99.6 (98.7–100.0)	0.001	16.7 (0.0–37.8)	96.1 (90.8–100.0)	0.014
15%	33.3 (0.0–86.7)	99.6 (98.7–100.0)	0.003	25.0 (0.0–55.0)	96.4 (91.4–100.0)	0.015
20%	50.0 (0.0–100.0)	99.6 (98.7–100.0)	0.003	28.6 (0.0–62.0)	96.4 (91.6–100.0)	0.012
25%	50.0 (0.0–100.0)	99.6 (98.7–100.0)	0.003	28.6 (0.0–62.0)	96.4 (91.6–100.0)	0.005
30%	100.0 (100.0–100.0)	99.6 (98.7–100.0)	0.004	50.0 (1.0–99.0)	96.6 (92.0–100.0)	0.018

*Note:* Net Benefit (NB) > 0 indicates added clinical value compared with the Two‐Step Strategy.

^a^ In the Two‐Step Strategy, all participants in the subgroup meeting a modified Benign Descriptor (BD) criteria received a fixed malignancy risk of 1%, resulting in no positive tests for thresholds ≥ 1%. Consequently, PPV is undefined and NPV is constant at 99.2% (98.0–100.0) in the non‐tertiary centres.

^b^ Net Benefits (NB) are not reported for the Two‐Step Strategy, as the decision curve coincides with the reference strategies (treat‐none for thresholds > 1%, treat‐all for thresholds ≤ 1%).

Excluding pregnant participants (*n* = 17/966, 1.8%), among whom no malignancies were observed, did not affect model performance. Differences from the primary analysis were minimal (ΔAUC ≤ 1.0 pp, ΔNPV ≤ 0.2 pp, ΔPPV ≤ 1.2 pp, ΔBrier ≤ 0.001).

## 4 Discussion

This study provides a large‐scale, prospective evaluation of the ADNEX model and the Two‐Step Strategy with modified BDs in routine gynaecologic care in Denmark, including both tertiary and non‐tertiary settings, ultrasound examinations performed by clinicians (medical doctors) involved in patient management, and both surgically and conservatively managed patients. Model performance was evaluated separately in tertiary and non‐tertiary centres to account for differences in malignancy prevalence, and threshold analyses were focused on the non‐tertiary setting representing the pre‐triage population. Across all settings, the IOTA‐based models demonstrated higher discriminative ability than RMI and consistently identified a greater proportion of malignancies. In non‐tertiary centres, lower risk thresholds were associated with fewer missed cancers but higher referral rates, illustrating the trade‐off between sensitivity and resource utilization. At higher thresholds, referral rates approached those of RMI, while the gain in malignancy detection was modest. For example, at a 10%–15% risk threshold in non‐tertiary centres, the IOTA‐based models identified approximately 63%–72% of malignancies compared with 39% using RMI, corresponding to 10–15 additional detected cases per 100 women with malignancy, at the cost of increased referrals. Predictive values varied markedly between tertiary and non‐tertiary settings and should be interpreted in the context of underlying malignancy prevalence. Most additionally detected tumours were borderline and stage I OC, indicating improved detection of early‐stage disease, whereas differences in advanced‐stage OC and metastases were limited. Calibration was overall acceptable, with minor underestimation in the clinically relevant risk range of approximately 5%–20%. Lower thresholds of the IOTA models favoured sensitivity and early detection, whereas higher thresholds reduced false positives and referral burden. Thresholds ≥ 25% resulted in referral rates comparable to RMI ≥ 200 but yielded only limited improvement in malignancy detection. The Two‐Step Strategy effectively excluded malignancy using the modified BD, and applying the ADNEX model within this subgroup provides minimal additional diagnostic value.

Evidence on the diagnostic performance of IOTA‐based models in real‐world settings has been limited, particularly in general cohorts from only non‐tertiary centres where the triage models are intended for use, with non‐expert examiners performing ultrasound examination, and including conservatively managed patients. The AUCs of our study population overall combining non‐tertiary and tertiary centres were comparable to the recently published Moro et al. [24] and Lems et al. [25] who both reported AUCs around 0.91–0.92. The higher PPVs reported by Moro et al. [24] (PPV 31%–80% vs. 24.4%–63.7% in ours) is likely explained by their exclusively surgically managed population (30.5% malignancy vs. ~21% in ours). In line with Lems et al. [25], missed malignancies were mainly borderline ovarian tumours and stage I OC. Moro et al. [24] found stable model performance across oncology centres (AUC: RMI 0.85, ADNEX 0.92, Two‐Step 0.92) and slightly higher AUCs in non‐oncology centres. In contrast, we observed lower AUCs across all models, particularly in the tertiary centre. This may be related to differences in case mix, as our cohort included a higher proportion of histologically verified endometriomas (16% vs. 14%) as well as conservatively managed suspected endometriomas, which may increase overlap between benign and malignant risk profiles. The lower AUCs in non‐oncology centres may also be influenced by the small number of malignant cases and resulting in imprecision of estimates. The UK ROCkeTS study by Sundar et al. [26] similarly reported comparable AUCs (0.89) for ADNEX in a mixed secondary‐care setting of exclusively postmenopausal participants, supporting our results.

Model and threshold selection should be guided by clinical priorities and healthcare capacity. For a rare but fatal disease like OC, where early identification and referral to specialized gynaecologic oncology centres substantially improve prognosis in advanced‐stage disease, a high NPV may be more clinically valuable than a high PPV. Lower thresholds favour early detection but increase false‐positive referrals. A recent model‐based cost–consequence analysis also highlighted the trade‐offs between diagnostic yield, healthcare costs, and cancer outcomes, suggesting that IOTA‐based strategies may offer a more balanced approach compared with traditional methods such as RMI [34]. While such strategies may be acceptable in high‐resource settings, they may strain specialist services, prolong waiting times, and expose patients to unnecessary interventions and anxiety [5]. A recent meta‐analysis and consensus report proposed a tiered approach: conservative management for risk < 1%, surgery in general gynaecology units for risk < 10%, and referral to oncology centres for risk ≥ 10% [15, 19]. In our cohort, no malignancies were missed at the 1% threshold. Internationally, recommendations vary: the Netherlands has adopted a 40% ADNEX threshold [35], Lems et al. suggested 7%–14% as Youden‐optimal range (statistically maximizing sensitivity and specificity with equal weighting) [25], and the ROCkeTS study supported 10% in postmenopausal women [26]. In our study, thresholds around ≥ 25% produced referral rates like RMI but offered only limited additional diagnostic gain.

The Two‐Step Strategy is increasingly advocated as an alternative [21, 23, 24], sparing CA125 testing when a modified BD applies. In our cohort, the ADNEX model added minimal diagnostics value in this subgroup by detecting a few borderline tumours, though at the cost of more false positives. While borderline tumours generally have favourable prognosis and limited survival benefit from earlier detection compared with primary OC, correct preoperative classification and referral may still have important clinical implications. In the Danish setting, patients with borderline tumours undergo surgical staging according to national guidelines, and referral to a tertiary centre at the primary operation may reduce the need for secondary staging procedures. This may decrease surgical morbidity, avoid repeated interventions, and improve care pathways. These considerations should be weighed against the increased referral burden when interpreting the clinical value of additional detections. Overall, the Two‐Step Strategy showed similar performance to ADNEX, suggesting that its main advantage lies in simplifying the diagnostic workflow rather than improving detection.

Our study has several strengths. The cohort included both pre‐ and postmenopausal patients, managed surgically and conservatively, with ultrasound examinations performed by clinicians of varying experience and IOTA certification status, representing Danish clinical practice. CA125 was available for nearly all participants, and most samples were processed under standardized biobank protocols. Diagnostic performance was stratified by centre type to account for differences in malignancy prevalence between non‐tertiary versus tertiary settings. This allowed evaluation of model performance across clinically relevant contexts and ensured that threshold analyses were based on the non‐tertiary cohort representing the intended pre‐triage population. Several limitations should be considered. Ultrasound examinations were performed without centralized quality control, and variables were based on clinician‐reported findings without central review. Undocumented features were assumed to be absent. These factors may have introduced variability and misclassification of ultrasound features, potentially influencing model performance. Modified BD applicability and ADNEX scores were derived retrospectively from prospectively recorded ultrasound data rather than computed at the point of care. In addition to limiting resemblance to real‐time clinical practice, this approach may have introduced data abstraction errors and misclassification of input variables. The number of malignant cases in non‐tertiary centres was limited, resulting in wider confidence intervals and less stable threshold‐specific estimates. Recruitment was initiated at a tertiary centre during the COVID‐19 period and subsequently expanded to non‐tertiary sites, which may have introduced temporal heterogeneity in case mix and referral patterns. Calendar time was not formally evaluated as a potential confounder. As recruitment was not strictly consecutive and only patients who agreed to participate were recorded, selection bias cannot be excluded. A complete‐case analysis was performed, and although tumour distribution was similar between included and excluded participants, residual selection bias cannot be ruled out. Inclusion of both general gynaecology practices and hospital‐based departments may have introduced heterogeneity in case mix and diagnostic practice, although these settings represent integrated components of the Danish healthcare system and reflect routine clinical pathways. The use of different reference standards for surgically and conservatively managed patients may have introduced partial verification bias. Patients undergoing surgery had histological confirmation and were more likely to have a higher baseline risk of malignancy, whereas conservatively managed patients were classified based on clinical follow‐up and registry data. Although no malignancies were identified in the conservatively managed group, the absence of histological verification may have led to underestimation of missed malignancies, potentially affecting sensitivity estimates. However, in clinical practice, patients with suspected advanced malignancy or those deemed inoperable typically undergo biopsy or cytological verification and were therefore included in the surgically managed group, which may have reduced the extent of this bias. CA125 measurements were obtained either at inclusion or from recent routine clinical testing, which may have introduced variability due to temporal changes in biomarker levels. A small number of pregnant participants were included, although the ADNEX model has not been specifically validated in this population. Sensitivity analyses excluding these patients did not materially affect the results.

Because RMI ≥ 200 guided triage to the tertiary centre, participants in this setting were enriched for RMI‐positive cases, introducing spectrum bias. In addition, as ADNEX and the Two‐Step Strategy were calculated retrospectively and did not influence clinical decision‐making, the comparison represents a triage‐guided pathway versus a hypothetical alternative applied post hoc. This may limit the internal validity of direct comparisons between models. The generalizability of our findings may be influenced by the structure of the Danish healthcare system, which includes centralized OC surgery and well‐defined referral pathways. In settings without similar infrastructure, the balance between detection and referral burden, as well as the clinical impact of implementing IOTA‐based models, may differ.

In conclusion, this evaluation in routine gynaecologic care suggests that IOTA‐based models may be considered as alternatives to RMI for triaging adnexal masses in Denmark. Given the aggressiveness of OC and the survival benefit of early‐stage detection, the diagnostic gain is likely to outweigh the increase in referrals. However, implementation should be adapted to available healthcare capacity and clinical context. The Two‐Step Strategy appeared more efficient than the ADNEX model alone, achieving similar detection rates with fewer referrals, indicating limited additional diagnostic value of ADNEX in this setting. At a 15% risk threshold in non‐tertiary settings, the ADNEX model would detect approximately 19 additional malignancies per 1000 women evaluated compared with RMI, at the cost of approximately 68 additional referrals. This illustrates the trade‐off between improved detection and increased referral rates. A threshold around 15% may therefore represent a clinically relevant balance between minimizing false negatives and avoiding excessive referrals, although the optimal threshold should be adapted to local healthcare capacity and priorities. If resources are limited, a 20% threshold may be acceptable, though at the cost of reduced detection. An implementation audit is recommended to fine‐tune the optimal threshold based on local outcomes and capacity. These findings may inform future health economic and implementation studies evaluating the impact on referral patterns, resource use, and clinical outcomes, which are needed to guide potential changes in clinical guidelines.

## Author Contributions

N.S.K., E.D., A.E.S., M.A.K., E.S.H. and C.K.H. conceived and designed the study, developed the methodology, and contributed to protocol development and approvals. N.S.K., E.D., A.E.S., M.A.K. and C.K.H. coordinated recruitment at Rigshospitalet. E.S.H. managed blood handling, storage and biomarker material distribution. N.S.K. coordinated the study. L.L.‐T., M.M.F. and G.A. coordinated implementation at collaborating hospitals. N.S.K. and T.A.G. performed statistical analyses. All authors reviewed the results, revised the manuscript, and approved the final version for submission.

## Funding

N.S.K.'s PhD position was funded by the Danish Cancer Society and the NEYE Foundation. Roche Diagnostics A/S (Denmark) donated the assay kits used for CA125 analyses in biobank samples; CA125 is an established biomarker in routine clinical practice. Roche Diagnostics had no role in the study design, data collection, analysis, interpretation of findings, or the decision to publish the results.

## Ethics Statement

The study was approved by the Regional Ethics Committee of the Capital Region of Denmark (journal no. H‐19021342, approved 12 July 2019).

## Consent

Written and oral informed consent was obtained from all participants.

## Conflicts of Interest

The authors declare no conflicts of interest.

## Supporting information


**Figure S1:** Calibration curves for the ADNEX model and Two‐step strategy.
**Table S1:** Distribution of malignant tumour types detected at different thresholds of the ADNEX model, Two‐Step Strategy and RMI among participants enrolled at non‐tertiary centres.
**Table S2:** Distribution of malignant tumour types detected at different thresholds of the ADNEX model, Two‐Step Strategy and RMI among participants enrolled at the tertiary centre.
**Table S3:** Diagnostic performance of the ADNEX model and the Two‐Step Strategy across selected risk thresholds in the sensitivity analysis, including examinations from non‐oncology care settings (non‐tertiary centres and the specialized tertiary unit for complex endometriosis).

## Data Availability

The data that support the findings of this study are available on request from the corresponding author. The data are not publicly available due to privacy or ethical restrictions.
