# Supplementary material for: Evaluation of the IOTA ADNEX Model, Two‐Step Strategy and RMI in Routine Gynaecologic Care in Denmark and Implications for Implementation: A Prospective Multicenter Cohort Study
Source: BJOG. 2026 Apr 26;133(11):2013–24. doi: 10.1111/1471-0528.70253 (PMC13571888; doi:10.1111/1471-0528.70253)
Supplement: Supplementary file 1 [file BJO-133-2013-s001.docx]

**All supplementary material:**

**Table S1. Distribution of malignant tumor types detected at different thresholds of the ADNEX model, Two-Step Strategy, and RMI among participants enrolled at non-tertiary centers.** Values are n/N (%) where n is the number detected above the decision threshold (or RMI ≥ 200) and N is the total number of that subtype. “Total detected malignancies” is the number detected across all malignant subtypes relative to all malignant cases (N = 44).

| < threshold | Model | Detected malignancies by type (n/N, %) at the non-tertiary centers | | | | |
| --- | --- | --- | --- | --- | --- | --- |
|  |  | **Borderline** | **Stage I OC** | **Stage II-IV OC** | **Metastases to the ovary** | **Total detected malignancies** |
| 1 % | ADNEX  Two-step strategy | 23/23 (100.0%)  23/23 (100.0%) | 9/9 (100.0%)  9/9 (100.0%) | 7/7 (100.0%)  7/7 (100.0%) | 5/5 (100.0%)  5/5 (100.0%) | 44/44 (100.0%)  44/44 (100.0%) |
| 5 % | ADNEX  Two-step strategy | 21/23 (91.3%)  20/23 (87.0%) | 8/9 (88.9%)  8/9 (88.9%) | 7/7 (100.0%)  7/7 (100.0%) | 3/5 (60.0%)  3/5 (60.0%) | 39/44 (88.6%)  38/44 (86.4%) |
| 10 % | ADNEX  Two-step strategy | 16/23 (69.6%)  15/23 (65.2%) | 7/9 (77.8%)  7/9 (77.8%) | 6/7 (85.7%)  6/7 (85.7%) | 3/5 (60.0%)  3/5 (60.0%) | 32/44 (72.7%)  31/44 (70.5%) |
| 15 % | ADNEX  Two-step strategy | 15/23 (65.2%)  14/23 (60.9%) | 5/9 (55.6%)  5/9 (55.6%) | 5/7 (71.4%)  5/7 (71.4%) | 3/5 (60.0%)  3/5 (60.0%) | 28/44 (63.6%)  27/44 (61.4%) |
| 20 % | ADNEX  Two-step strategy | 14/23 (60.9%)  13/23 (56.5%) | 4/9 (44.4%)  4/9 (44.4%) | 5/7 (71.4%)  5/7 (71.4%) | 3/5 (60.0%)  3/5 (60.0%) | 26/44 (59.1%)  25/44 (56.8%) |
| 25 % | ADNEX  Two-step strategy | 12/23 (52.2%)  11/23 (47.8%) | 3/9 (33.3%)  3/9 (33.3%) | 5/7 (71.4%)  5/7 (71.4%) | 3/5 (60.0%)  3/5 (60.0%) | 23/44 (52.3%)  22/44 (50.0%) |
| 30 % | ADNEX  Two-step strategy | 11/23 (47.8%)  10/23 (43.5%) | 3/9 (33.3%)  3/9 (33.3%) | 5/7 (71.4%)  5/7 (71.4%) | 3/5 (60.0%)  3/5 (60.0%) | 22/44 (50.0%)  21/44 (47.7%) |
|  | | | | | | |
| 200 | RMI | 8/23 (34.8%) | 3/9 (33.3%) | 4/7 (57.1%) | 2/5 (40.0%) | 17/44 (38.6%) |
| Denominators for percentages are based on all malignant cases in non-oncology centers (23 borderline, 9 stage I OC, 7 stage II–IV OC, 5 metastases). | | | | | | |

**Table S2. Distribution of malignant tumor types detected at different thresholds of the ADNEX model, Two-Step Strategy, and RMI among participants enrolled at the tertiary center.** Values are n/N (%) where n is the number detected above the decision threshold (or RMI ≥ 200) and N is the total number of that subtype. “Total detected malignancies” is the number detected across all malignant subtypes relative to all malignant cases (N = 166).

| < threshold | Model | Detected malignancies by type (n/N, %) at the tertiary center | | | | |
| --- | --- | --- | --- | --- | --- | --- |
|  |  | **Borderline** | **Stage I OC** | **Stage II-IV OC** | **Metastases to the ovary** | **Total detected malignancies** |
| 1 % | ADNEX  Two-step strategy | 48/48 (100.0%)  48/48 (100.0%) | 27/27 (100.0%)  27/27 (100.0%) | 66/66 (100.0%)  66/66 (100.0%) | 25/25 (100.0%)  25/25 (100.0%) | 166/166 (100%)  166/166 (100%) |
| 5 % | ADNEX  Two-step strategy | 47/48 (97.9%)  44/48 (91.7%) | 26/27 (96.3%)  26/27 (96.3%) | 65/66 (98.5%)  65/66 (98.5%) | 24/25 (96.0%)  24/25 (96.0%) | 162/166 (97.6%)  159/166 (95.8%) |
| 10 % | ADNEX  Two-step strategy | 39/48 (81.2%)  37/48 (77.1%) | 25/27 (92.6%)  25/27 (92.6%) | 65/66 (98.5%)  65/66 (98.5%) | 24/25 (96.0%)  24/25 (96.0%) | 153/166 (92.2%)  151/166 (91.0%) |
| 15 % | ADNEX  Two-step strategy | 37/48 (77.1%)  35/48 (72.9%) | 24/27 (88.9%)  24/27 (88.9%) | 62/66 (93.9%)  62/66 (93.9%) | 23/25 (92.0%)  23/25 (92.0%) | 146/166 (88.0%)  144/166 (86.7%) |
| 20 % | ADNEX  Two-step strategy | 36/48 (75.0%)  34/48 (70.8%) | 24/27 (88.9%)  24/27 (88.9%) | 61/66 (92.4%)  61/66 (92.4%) | 20/25 (80.0%)  20/25 (80.0%) | 141/166 (84.9%)  139/166 (83.7%) |
| 25 % | ADNEX  Two-step strategy | 35/48 (72.9%)  33/48 (68.8%) | 24/27 (88.9%)  24/27 (88.9%) | 60/66 (90.9%)  60/66 (90.9%) | 19/25 (76.0%)  19/25 (76.0%) | 138/166 (83.1%)  136/166 (81.9%) |
| 30 % | ADNEX  Two-step strategy | 34/48 (70.8%)  32/48 (66.7%) | 24/27 (88.9%)  24/27 (88.9%) | 59/66 (89.4%)  59/66 (89.4%) | 19/25 (76.0%)  19/25 (76.0%) | 136/166 (81.9%)  134/166 (80.7%) |
|  | | | | | | |
| 200 | RMI | 24/48 (50.0%) | 17/27 (63.0%) | 62/66 (93.9%) | 21/25 (84.0%) | 124/166 (74.7%) |
| Denominators for percentages are based on all malignant cases in the tertiary center (48 borderline, 27 stage I OC, 66 stage II–IV OC, 25 metastases). | | | | | | |

**Table S3. Diagnostic performance of the ADNEX model and the Two-Step Strategy across selected risk thresholds in the sensitivity analysis, including examinations from non-oncology care settings (non-tertiary centers and the specialized tertiary unit for complex endometriosis).** Positive predictive value (PPV) and negative predictive value (NPV) are shown with 95% confidence intervals (CIs). *Above threshold* indicates the number and proportion of participants exceeding each threshold. *Malignant ≥ threshold* (n/N, %) represents the number and proportion of malignant tumors detected at or above each threshold (sensitivity).

|  | Non-tertiary centers and the specialized tertiary unit for complex endometriosis:  N = 684 (70.8%). Malignancies n = 49 (7.2%) | | | | |
| --- | --- | --- | --- | --- | --- |
| Thres-  hold ≥ | **Model** | **PPV (95% CI)** | **NPV (95% CI)** | **Above threshold,**  **n (%)** | **Malignant ≥ threshold,**  **n/N (% of all malignant)** |
| 1 % | ADNEX  Two-step strategy | 8.4 (6.1-10.6)  7.5 (5.5-9.5) | 100.0 (100.0-100.0)  100.0 (100.0-100.0) | 584 (85.4%)  652 (95.3%) | 49 (100%)  49 (100%) |
| 5 % | ADNEX  Two-step strategy | 18.1 (13.2-23.0)  19.7 (14.4-25.1) | 98.7 (97.6-99.7)  98.5 (97.4-99.6) | 238 (34.8%)  213 (31.1%) | 43 (87.8%)  42 (85.7%) |
| 10 % | ADNEX  Two-step strategy | 23.2 (16.4-29.9)  24.1 (17.1-31.2) | 97.4 (96.0-98.7)  97.2 (95.9-98.6) | 151 (22.1%)  141 (20.6%) | 35 (71.4%)  34 (69.4%) |
| 15 % | ADNEX  Two-step strategy | 28.7 (20.2-37.2)  28.8 (20.1-37.6) | 96.9 (95.5-98.3)  96.7 (95.3-98.2) | 108 (15.8%)  104 (15.2%) | 31 (63.3%)  30 (61.2%) |
| 20 % | ADNEX  Two-step strategy | 32.6 (22.8-42.3)  32.6 (22.7-42.5) | 96.6 (95.2-98.1)  96.5 (95.0-98.0) | 89 (13.0%)  86 (12.6%) | 29 (59.2%)  28 (57.1%) |
| 25 % | ADNEX  Two-step strategy | 37.1 (25.8-48.5)  37.3 (25.7-48.9) | 96.3 (94.8-97.8)  96.1 (94.6-97.6) | 70 (10.2%)  67 (9.8%) | 26 (53.1%)  25 (51.0%) |
| 30 % | ADNEX  Two-step strategy | 42.4 (29.8-55.0)  41.4 (28.7-54.1) | 96.2 (94.7-97.7)  96.0 (94.5-97.5) | 59 (8.6%)  58 (8.5%) | 25 (51.0%)  24 (49.0%) |
|  |  |  |  |  |  |
| 200 | RMI | 31.1 (19.9–44.3) | 95.2 (93.2–96.7) | 61 (8.9%) | 19 (38.8%) |
| Percentages in “≥ threshold” columns are relative to the total study population. Percentages in “Malignant ≥ threshold” columns are relative to all malignant tumors within each stratum (sensitivity). | | | | | |

**Figure S1. Calibration curves for the ADNEX model and Two-step strategy.**
